# Diagnosis of Fraser syndrome missed out until the age of six months old in a low-resource setting: a case report

**DOI:** 10.1186/s12887-019-1673-6

**Published:** 2019-08-22

**Authors:** Aimé Mbonda, Francky Teddy Endomba, Ulrick S. Kanmounye, Jan René Nkeck, Joel Noutakdie Tochie

**Affiliations:** 1 Department of Public Health, Faculty of Medicine and Biomedical Sciences, The University of Yaoundé I, Yaounde, Cameroon; 2 District Hospital Djohong, Adamawa, Cameroon; 3 Department of Internal Medicine, Faculty of Medicine and Biomedical Sciences, The University of Yaoundé I, Yaounde, Cameroon; 4 International Team, InciSioN, Adamawa, Cameroon; 5 Program in Global Surgery and Social Change, Harvard Medical School, Boston, USA; 6 Faculty of Medicine, Université Technologique Bel Campus, Kinshasa, DR Congo; 7 Department of Surgery and Anaesthesiology, Faculty of Medicine and Biomedical Sciences, University of Yaoundé I, Yaounde, Cameroon

**Keywords:** Fraser syndrome, Cryptophthalmos, Syndactyly, Anal imperforation, Cameroon

## Abstract

**Background:**

Fraser syndrome is a rare genetic disorder that often presents with ocular, renal, genital and limb’s congenital anomalies. The prognosis of this genetic disorder depends on the severity of the combination of congenital malformations, some of which may be fatal. The diagnosis of Fraser syndrome is based on established clinical criteria and genetic tests. The criteria enabling clinical diagnosis are visible dysmorphic features present at birth, hence, Fraser syndrome can easily diagnosed at birth, except when health professionals are inexperienced in clinical recognition. Herein, we report a case of Fraser syndrome missed out at birth and fortuitously diagnosed at the age of six months in a bid to raise clinicians’ awareness, particularly in resource-limited settings.

**Case presentation:**

We report a case of a six-month-old Cameroonian female infant, born at home and taken the following day to a primary healthcare facility for neonatal care. Her mother had no antenatal care until birth. She presented at our health center with respiratory distress and fever. She had a temperature of 38.8 °C and signs of left lung basal consolidation, suggestive of a left lower lober pneumonia, confirmed on chest x-ray. Other incidental clinical findings were several dysmorphic features like bilateral cryptophthalmos, nasal malformation, anal imperforation (with a perianal fistula), an external genital anomaly and syndactyly characteristic of Fraser syndrome associated with pneumonia. The patient responded well to intravenous antibiotics for the treatment of her pneumonia. Thereafter, she was referred to a pediatric surgeaon for surgical corrections of her bilateral cryptophthalmos, anal imperforation, external genital defect and syndactyly.

**Conclusion:**

Here we presented a case of Fraser syndrome in a Cameroonian infant whose diagnosis was missed out at birth and fortuitously made at six months of age. In view of the serious and potentially fatal complications of this genetic disorder, we draw clinicians’ attention, especially obstetricians, midwives and pediatricians for a high index of clinical suspicion geared at a timely diagnosis and management. Also, for a timely diagnosis, health education on regular antenatal and postnatal follow ups of  the mother-infant couple respectively, cannot be overemphasized.

## Background

Also known as Fraser-Francois syndrome, Meyer-Schwickerath’s syndrome, Ulrich-Feichtiger syndrome or cryptophthalmos-syndactyly syndrome, or simply Fraser syndrome, this syndrome was first described by Pliny the Elder, and first published in 1962 by a Canadian geneticist named George R. Fraser [1, 2]. It is a rare and autosomal recessive disease, with an incidence below 0.043 per 10,000 live births and 1.1 per 10,000 stillbirths [3]. Fraser syndrome is characterized by several congenital malformations such as cryptophthalmos, syndactyly, ear anomalies, respiratory tract malformations, and urogenenital anomalies [2, 4, 5]. Fraser syndrome is genetically distinguished by several mutations caused by the FRAS1, FREM1, FREM2 and GRIP1 genes essentials for the adhesion between the basal membrane and connective tissues during the embryonic period [5–7].

Cryptophthalmic eyes, with failure of eyelid formation, is the most common clinical feature of Fraser syndrome. The diagnosis of Fraser syndrome is made in the presence of one major criterion such as cryptophthalmos, syndactyly, anal imperforation, external genitalia, limb anomalies, and one minor criterion such as nasal, laryngeal or ear malformations, skeletal defects, umbilical hernia, mental retardation, respiratory and urogenital tract anomalies [8, 9]. Alternatively, a patient with Fraser syndrome can be diagnosed based on two of the major criteria and one minor criterion or one major criterion and four minor criteria mentioned above [9]. The disease can be suspected during the prenatal period by ultrasound detection of related malformations’ signs, and nearly 25% of foetuses with Fraser syndrome are delivered stillborn [2, 5]. The prognosis of survivors greatly depends on the overall combination of malformations which may require surgery for a definitive cure [2, 5]. Herein, we present a case of Fraser syndrome with several visible malformations overlooked at birth and diagnosed fortuitously at the age of six months when the infant was sick of another disease.

## Case presentation

A six-month-old female infant living in urban Yaoundé, Cameroon presented to our emergency department with a fever, difficulties in breathing and refusal to feed, all of progressive onset. These symptoms lasted 48 h before consultation, and no medication was given to the infant before her presentation. Her past history was relevant for several points. Firstly, her mother did not attend any antenatal care visit during pregnancy. However, her mother reported she had vaginal delivery at home at nine months of pregnancy without professional care. The infant had a vigorous cry at birth. The mother of the child sought neonatal and postpartum care at day one postpartum in a nearby health center where the neanate's diagnosis was surprisingly overlooked. She was the first child born to a 29-year-old HIV woman, with histories of psychiatric disorders and bilateral club foot. We could not have more information on the father of the infant because he abandoned her mother during her pregnancy. However, there was no consanguinity between both parents. There was no contact history of passive smoking and/or acute, sub-acute or chronic cough and her psychomotor development were adequate for her age. There was no family history of similar congenital malformations.

**Fig. 1:**
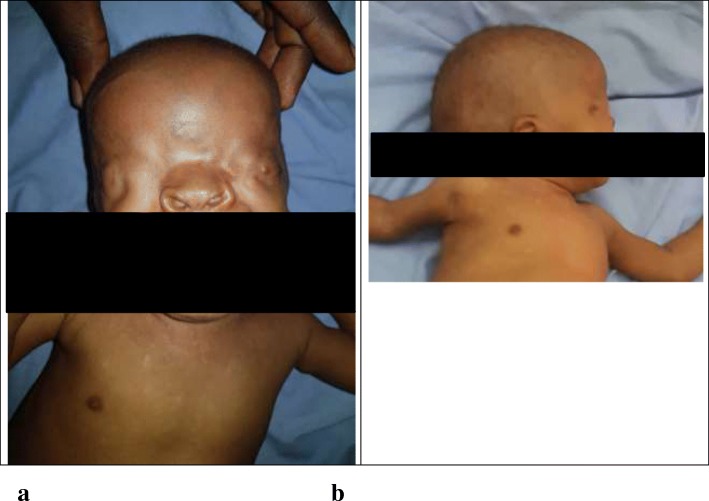
The infant with Fraser syndrome illustrating bilateral cryptophtalmos (**a** and **b**) and nasal malformation (**b**)

**Fig. 2:**
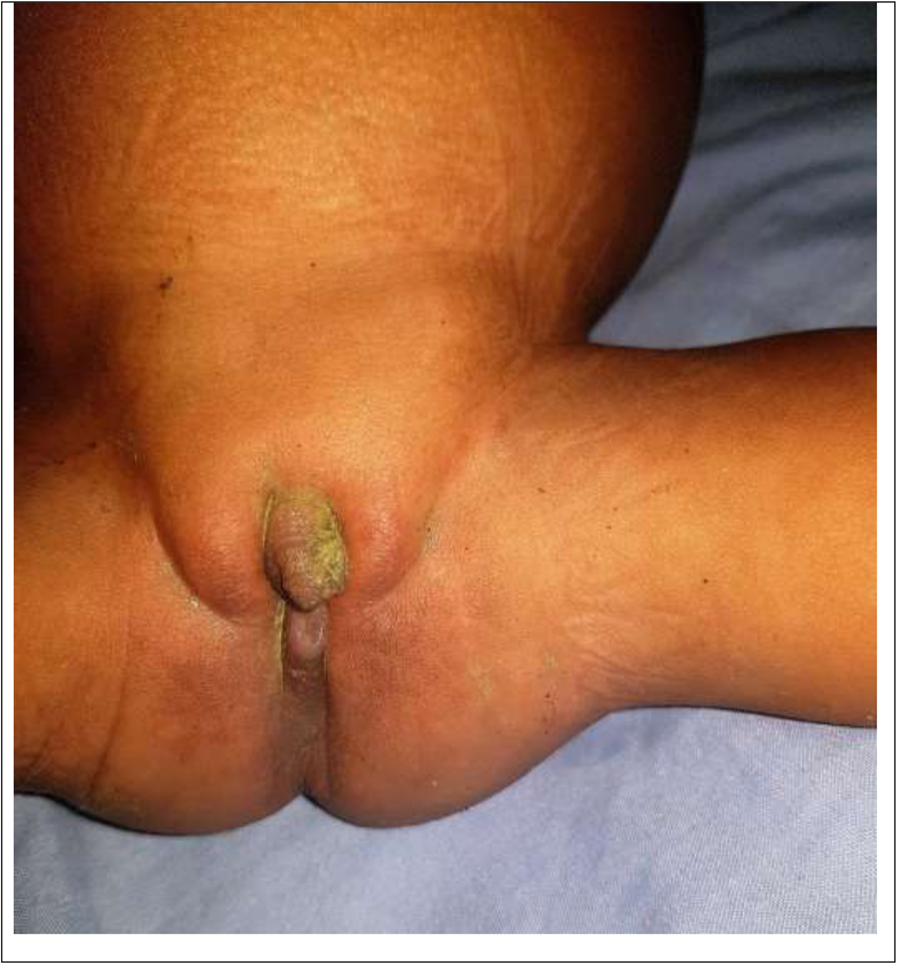
The infant with Fraser syndrome illustrating an external genitalia anomaly

On physical examination of the infant at admission, she was conscious, ill-looking, had a temperature at 38.8 °C, heart rate of 108 beats per minute, a respiratory rate (RR) of 42 cycles per minute and a peripheral oxygen saturation of 89% at ambient air. She had the following dysmorphic features: bilateral cryptophthalmos (Fig. 1), syndactyly, nasal malformation, anal imperforation with a nearby anal fistula (where she passes out feces) and an external genitalia anomaly (Fig. 2). She had no sign of dehydration. Respiratory examination showed signs of respiratory distress (tachypnoea, nasal flaring and chest recessions) and signs of consolidation of the base of the a left lung. Confronted with these dysmorphic features, they prompted a quick diagnostic review focusing on her clinical features, thereby advocating the diagnosis a community acquired pneumonia associated with Fraser syndrome in a six-month-old infant. The differential diagnosis thought of was Manitoba oculotrichonal syndrome. A chest x-ray showed a confluent homogenous opacity of the lower lobe of the left lung, suggestive of pneumonia. A laboratory panel requested on her admission revealed complete blood count: white blood cell count (WBC) 16,700/mm^3^ with neutrophilia (13,000/mm^3^ or 78%), haemoglobin 11.8 g/dl, platelet count of 238,000/mm3; random capillary blood glucose 92 mg/dl, and raised C-reactive proteins (CRP) at 96 mg/l. Her blood and urine cultures were sterile. She was hospitalised in the pediatric department and treated with amoxicillin-clavulanic acid 100 mg/kg/24 h intravenously (IV) in three divided doses, paracetamol 15 mg/kg/06 h IV. She was equally administered oxygen through nasal prongs at 3 l/min.

On the second day of hospitalization, she had no fever and signs of respiratory distress but was still ill-looking. On the fifth day of hospitalization she had a good general state. Repeat blood tests showed normal WBC 10,900/mm^3^, haemoglobin 12 g/dl, thrombocytosis 298,000/mm^3^, and CRP negative (less than 6 mg/l). At the 10th day of hospitalization, she had no fever, no respiratory distress and was not ill-looking. Our health facility not being equipped with a peadiatric surgical unit, she was referred to the paediatric surgical department of the Yaounde Obstetric and Paediatric Hospital in Cameroon for surgical management of her bilateral cryptophthalmos, syndactyly, nasal malformation, anal imperforation and ambiguous genitalia external.

## Discussion and conclusion

We presented the case of a six-month-old infant lately diagnosed with Fraser syndrome. Indeed, as previously said, the diagnosis of Fraser syndrome is made base on clinical criteria advocated by Thomas et al. in 1986 [9]. These include the presence of one major and minor clinical criteria or two major criteria and one minor criterion or one major criterion and at least four minor criteria [8, 9]. Hence, our patient fulfilled the case definition of Fraser syndrome by presenting with three major criteria: cryptophthalmos, syndactyly, and external genitalia anomaly and two minor criteria: nasal malformation, and external genitalia anomaly.

Cryptophthalmos ocuurs in 93% of infants with Fraser syndrome and it is due to a failure of eyelid formation [10]. It represents a primitive failure in the surface of embryologic ectoderm, between 6th and 8th weeks of gestation [8, 11, 12]. The exact origin of this syndrome remains unclear but the pathogenesis has been hypothesized to be linked to a failure of the programmed cell necrosis or defects in epidermal adhesion [8, 11, 12]. Also, some genetic aberrations on the FRAS1, FREM1, FREM2, and GRIP1 genes have been found in about 50% of cases of Fraser syndrome [5–7]. Other gene mutations hypothesized to play a role in the pathogenesis of Fraser syndrome based on animal studies but not human studies include Hemicentin 1 (HMCN1), Furin and Fibrillin 2 gene. Noteworthy, FREM1 gene mutations are known to cause diseases of phenotypic variants different from Fraser Syndrome such as bifid nose with or without renal anomalies, Manitoba oculotrichonal syndrome [13], Trigonocephaly 2. Manitoba oculotrichonal syndrome differs dysmorphically from Fraser syndrome by the presence of a bifid nose, hypertelorism (spaced eyes) and microphthalmia in the former [13]. Like Fraser syndrome, infants with Trigonocephaly 2 also have mental retardation due to FREM1 gene mutation. But infants with Trigonocephaly 2 have triangularly shaped head unlike those with Fraser syndrome.

One other etiologic factor of Fraser syndrome is consanguinity. A European study found a high prevalence rate (27%) of consanguinity in families with Fraser syndrome [5]. By contrast, in the indexed case there was no history of consanguinity among the parents of the child.

The peculiarity in our case is that the diagnosis of Fraser syndrome was missed out at birth and only fortuitously diagnosed concomitantly when the child presented for a respiratory infection. Majority of cases of Fraser syndromes are diagnosed at birth or within the neonatal period. But the health care providers of the health facility where the mother sought neonatal and postpartum care after home delivery overlooked the diagnosis of Fraser syndrome within the neonatal period probably due to inexperience in the recognition of congenital malformation syndromes in our setting. Furthermore, the several visible malformations of the infant did not call the attention of her mother to seek a pediatric consult, probably due to her history of psychiatric disorder or due to the stigmatization of her HIV status.

It is possible to make a prenatal diagnosis of Fraser syndrome as early as the 18th week of pregnancy through an ultrasound scan which may show ambiguous genitalia, limb anomalies, and umbilical hernia related to the malformations seen in Fraser Syndrome [4, 5, 12]. Moreover, quantitative abnormalities of the amniotic fluid can be suggestive of respiratory tract and/or urogenital tract malformations [10]. In the present case, the diagnosis was missed antenatally due to a poor pregnancy follow up, without an obstetrical ultrasound scan done. It is worth to mention, that the infant’s mother had HIV infection. With an extensive literature search, to the best of our knowledge, there is currently no study in favor of an associatio between HIV infection or highly antiretroviral treatment in pregnancy and the occurrence of Fraser syndrome in foetuses or newbrons of the HIV infected mother.

The prognosis of infants with Fraser syndrome depends on the severity of the congenital malformations, especially respiratory tract malformations and death within the perinatal period or the first year of life is common [5, 8, 14]. Plausible explanations to the survival of this child until the age of six months when she presented with pneumonia could be due to the absence of life-threatening malformations like pulmonary malformations and anal imperforation.

Treatment options for Fraser syndrome are currently limited despite progress made in terms of the field of genetics. The global recommendation is surgical management depending on the malformations present (anal imperforation, syndactyly, cryptophthalmos, limb and skeletal defects, ambiguous external genitalia, respiratory tract or uro-genital anomaly), psychological support and genetic counseling for parents [2, 5, 15]. Hence, the above case was referred to a pediatric surgeon in order to evaluate the feasibility of the surgical management of cryptophthalmos, anal imperforation, syndactyly and external genitalia.

The absence of genetic analysis in the current report in search of FRAS1, FREM1, FREM2, and GRIP1 genes mutations to further affirm the diagnosis of Fraser syndrome is an important limitation of the present report. This ilustrates frequent diagnostic challenges encountered in poor-resourse settings [16–19]. However, it is worth to mention that till present date, genetic tests are not yet available in Cameroon.

In conclusion, Fraser syndrome can be diagnosed antenatally with ultrasonography and postnatally with a thorough clinical examination. This emphasizes the importance of adequate antenatal and postnatal follow up of pregnant women in our poor-resource setting. Unfortunately, most pregnant Cameroonian women cannot afford healthcare due to financial constraints. Hence, the adoption of a universal access to health may go a long way to help this vulnerable population of pregnant women. Furthermore, there is an urgent need for organizing refresher courses on recognition of congenital malformation syndromes at birth by healthcare professional especially obstetricians, midwives and pediatricians for a timely diagnosis this potentially fatal genetic disorder and subsequent referral to a pediatric surgical department for prompt management geared at averting fatallife-threathening complications outcomes of Fraser syndrome.

## Data Availability

Data sharing is not applicable to this article as no datasets were generated or analysed during the current study.

## Abbreviations

CRP: c-reactive proyeins
IV: Intravenously
WBC: White blood cell count
